# Association Between Dietary Patterns and Myopia Among Children and Adolescents: A School‐Based Cross‐Sectional Study

**DOI:** 10.1155/joph/3892394

**Published:** 2026-02-19

**Authors:** Tongtong Li, Jing Yang, Jing Yan, Xuyang Yao, Bei Du, Qi Wu, Xiangda Meng, Yuanyuan Liu, Yuezhu Lu, Fei Ma, Yun Zhu, Qihua Wang, Qiang Yang, Chea-Su Kee, Cheuk Sing Jason Yam, Allen M. Y. Cheong, Ruihua Wei, Guowei Huang, Hua Yan

**Affiliations:** ^1^ School of Public Health, Tianjin Medical University, Tianjin, China, tijmu.edu.cn; ^2^ Tianjin Key Laboratory of Ocular Trauma, Tianjin, China; ^3^ Department of Ophthalmology, Tianjin Medical University General Hospital, Laboratory of Molecular Ophthalmology, Tianjin Medical University, Tianjin, China, tijmu.edu.cn; ^4^ Tianjin Key Laboratory of Retinal Functions and Diseases, Tianjin Branch of National Clinical Research Center for Ocular Disease, Eye Institute and School of Optometry, Tianjin Medical University Eye Hospital, Tianjin, China, tmuec.com; ^5^ Department of Ophthalmology, Peking Union Medical College Hospital, Peking Union Medical College, Chinese Academy of Medical Sciences, Beijing, China, cacms.ac.cn; ^6^ School of Clinical Medicine, Beijing Tsinghua Changgung Hospital, Tsinghua University, Beijing, China, tsinghua.edu.cn; ^7^ Shenyang Xingqi Pharmaceutical Co., Ltd, Shenyang, China; ^8^ School of Optometry, Centre for Myopia Research, Research Centre for SHARP Vision, The Hong Kong Polytechnic University, Hong Kong, China, polyu.edu.hk; ^9^ Department of Ophthalmology and Visual Sciences, The Chinese University of Hong Kong, Hong Kong, China, cuhk.edu.hk; ^10^ School of Optometry, The Hong Kong Polytechnic University, Hong Kong, China, polyu.edu.hk; ^11^ School of Medicine, Nankai University, Tianjin, China, nankai.edu.cn

**Keywords:** children, cross-sectional study, dietary pattern, FFQ, myopia

## Abstract

**Background:**

The myopia rate has increased rapidly worldwide, yet evidence regarding the association between dietary factors and myopia remains limited. This study assessed the association between dietary patterns and myopia among children and adolescents.

**Methods:**

This study used the Child and Adolescent Research of Eye data between August and October 2022. Myopia was defined based on uncorrected visual acuity and noncycloplegic refraction. Dietary assessment was parent‐reported via a food frequency questionnaire (FFQ). Principal component analysis was used to extract dietary patterns. Binary logistic regression was used to evaluate the association between dietary patterns and myopia.

**Results:**

A total of 24,797 participants were included in the analysis. Controlling for confounders, the highest adherence to nuts‐tubers vegetables pattern (characterized by high intake of nuts, tubers vegetables, legumes, whole grains, and aquatic products) was associated with a decreased risk of myopia compared with the lowest adherence (odds ratio [OR] = 0.933, 95% confidence interval [CI]: 0.872 to 0.999, *p* = 0.046). Conversely, the highest adherence to snacks pattern (characterized by high intake of fried and barbecued, fast foods and savoury snacks, sugar‐sweetened beverages, desserts, and processed meats) was associated with an increased risk of myopia (OR = 1.083, 95% CI: 1.012 to 1.158, *p* = 0.021).

**Conclusions:**

These findings indicate a link between dietary patterns and myopia in children and adolescents. Dietary modification could be a potential public health measure for the primary prevention of childhood myopia.

## 1. Introduction

Myopia is increasing at an alarming rate globally, particularly among school‐aged children in East Asia, and it is projected to affect half the world’s population by 2050 [1, 2]. The public health implications of this trend are significant; although any level of myopia predisposes individuals to adverse ocular changes, the risk of developing sight‐threatening conditions like glaucoma, retinal detachment, and macular atrophy increases substantially with high myopia [3]. In China, myopia prevalence rises sharply with school stage, reaching over 80% among senior high school students [4]. The early onset and rapid progression of myopia in children underscore the urgent need to identify modifiable risk factors for effective prevention.

Diet has emerged as a potential factor, but existing research offers conflicting results and has predominantly focused on single nutrients or specific foods. A 24‐week randomized controlled trial indicated that daily crocetin supplementation (a type of carotenoid) slowed the progression of spherical equivalent refraction (SER) and axial length elongation [5]. A cross‐sectional study conducted on Chinese children aged 6–12 years suggested that whole grains intake of > 50% could protect against myopia [6]. A cohort study assessed a wide range of dietary factors, but the associations between specific nutrients or food groups and myopia were not observed [7]. Evidence linking a wide range of dietary factors to myopia remains weak.

Given the above, this study extracted dietary patterns based on a wide range of food groups to assess the dietary factors comprehensively. Thus, this study aimed to explore the association between dietary patterns and myopia in children and adolescents based on Tianjin Child and Adolescent Research of Eye (TCARE) and to provide evidence regarding myopia prevention.

## 2. Materials and Methods

### 2.1. Study Population

**FIGURE 1:**
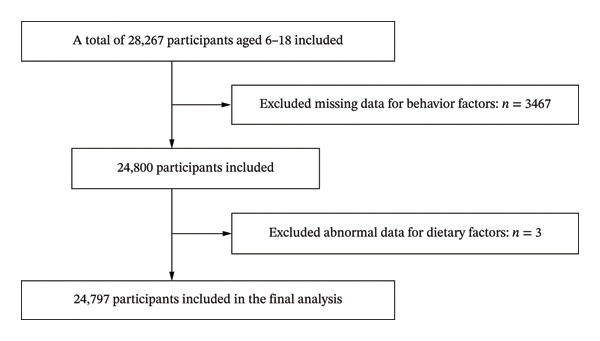
Flowchart of participants’ recruitment.

The TCARE, a large‐scale, citywide, and school‐based ocular screening, was conducted in Tianjin, China, from August to October 2022. A stratified, multistage, cluster sampling approach was employed for participant selection in this survey. Firstly, three districts were randomly selected from each of the three area types (rural, urban, and suburban) using probability proportional to size (PPS) sampling. Secondly, 20 primary schools, 10 junior high schools, and 6 senior high schools were randomly selected from each district, respectively. Thirdly, students from the second, fourth, sixth, eighth, and 11th grades were randomly chosen from each selected school. A total of 28,267 students between 6 and 18 years of age were enrolled. Among them, 3467 students with missing data for behavior factors and 3 students with abnormal data for dietary factors were excluded. Finally, 24,797 participants were analyzed, as described in Figure 1.

This study was conducted according to the guidelines laid down in the Declaration of Helsinki, and all procedures involving research study participants were approved by the Medical Ethics Committee of Tianjin Medical University Eye Hospital, China (no. 2020KY‐39), and the Ethical Committee of Tianjin Medical University General Hospital (no. IRB2022‐YX‐087‐01). Application for exemption from informed consent signature has been submitted to and approved by the Medical Ethics Committee of Tianjin Medical University Eye Hospital. All participants were informed of the research purpose through oral instruction. Verbal consent was witnessed and formally recorded.

### 2.2. Data Collection

Data on demographic characteristics, behavior, lifestyle, and parental myopia status were collected using the Child and Adolescent Behavior Questionnaire, which was completed by the parents. Demographic characteristics included age, sex, region, etc. Behavior and lifestyle mainly included homework time (< 1, 1–2, or ≥ 2 h/d) and outdoor exercise time (< 1, 1–2, or ≥ 2 h/d). For parental myopia, paternal myopia and maternal myopia were registered by questionnaire separately.

### 2.3. Ocular Examinations

Ocular examinations were conducted by certified ophthalmic practitioners who had received professional training. The standard logarithmic visual acuity E chart was used to examine uncorrected visual acuity (UCVA). SER was measured without cycloplegia with the Tianle RM‐9600 autorefractor (Shanghai, China). SER was calculated as sphere plus 0.5 cylinder. Students with SER ≤ −0.5 diopters (D) and UCVA < 5.0 in either eye were diagnosed with myopia [8].

### 2.4. Dietary Assessment

Average habitual dietary intake was parent‐reported via a self‐designed food frequency questionnaire (FFQ). Food items were categorized into 17 food groups based on similarities in nutrient composition or culinary methods: (1) refined grains; (2) whole grains; (3) leafy vegetables; (4) tubers vegetables; (5) fruits; (6) red meats; (7) poultry; (8) processed meats; (9) aquatic products; (10) eggs; (11) dairy products; (12) legumes; (13) nuts; (14) desserts and sweet snacks; (15) sugar‐sweetened beverages (SSB); (16) fast foods and savoury snacks; (17) fried and barbecued. Reporting options for the participant’s usual consumption frequency included “never” or the number of times “per week” or “per day”.

### 2.5. Statistical Analysis

All statistical analyses were performed using SPSS software (version 24.0). Categorical variables are presented as numbers and percentages, and differences between groups were compared using the chi‐square test or Wilcoxon’s rank sum test as appropriate. Principal component analysis (PCA) was used to extract dietary patterns based on 17 food groups in the FFQ. The data’s qualification for PCA was checked through the Kaiser–Meyer–Olkin (KMO) and Bartlett’s tests. Three dietary patterns were determined by factors with eigenvalues of more than 1.0, combined with the scree plot breakpoints and cumulative variance contributions. The factor scores were calculated using regression; higher scores indicate greater adherence to the respective dietary pattern. All participants were divided into T1, T2, and T3 according to the tertiles of the factor scores. Binary logistic regression was used to evaluate the association between dietary patterns and myopia. Odd ratios (ORs) and 95% confidence intervals (CIs) were calculated. The regression models were adjusted for age, sex, region, parental myopia, homework time, and outdoor exercise time. Statistical significance was considered when two‐tailed *p* < 0.05.

## 3. Results

### 3.1. General Characteristics of Study Participants

In total, 24,797 participants were included in this study; 12,962 (52.27%) were boys and 11,835 (47.73%) were girls. The basic characteristics of participants are shown in Table 1. Individuals with myopia were more likely to be female, 7–12th grade, living in suburban areas, parental myopia, longer homework time, and less outdoor exercise time (*p* < 0.001).

**TABLE 1:** Characteristics of participants with and without myopia (*n* [%]).

Characteristics	Total	Myopia		*p* ^a^
	(*n* = 24,797)	No (*n* = 10,728)	Yes (*n* = 14,069)	
Sex				< 0.001
Male	12,962 (52.27)	5881 (54.82)	7081 (50.33)	
Female	11,835 (47.73)	4847 (45.18)	6988 (49.67)	
Grade				< 0.001
1–6	16,736 (67.49)	9229 (86.03)	7507 (53.36)	
7–12	8061 (32.51)	1499 (13.97)	6562 (46.64)	
Region				< 0.001
Six central districts	14,918 (60.16)	6603 (61.55)	8315 (59.10)	
Four districts adjacent to the center	4443 (17.92)	2006 (18.70)	2437 (17.32)	
Suburb	5436 (21.92)	2119 (19.75)	3317 (23.58)	
Paternal myopia				< 0.001
No	13,831 (55.78)	6389 (59.55)	7442 (52.90)	
Yes	10,966 (44.22)	4339 (40.45)	6627 (47.10)	
Maternal myopia				< 0.001
No	13,058 (52.66)	6060 (56.49)	6998 (49.74)	
Yes	11,739 (47.34)	4668 (43.51)	7071 (50.26)	
Homework time (h/d)				< 0.001
< 1	3824 (15.42)	2296 (21.40)	1528 (10.86)	
1–2	17,465 (70.43)	7534 (70.23)	9931 (70.59)	
≥ 2	3508 (14.15)	898 (8.37)	2610 (18.55)	
Outdoor exercise time (h/d)				< 0.001
< 1	6672 (26.91)	2413 (22.49)	4259 (30.27)	
1–2	13,453 (54.25)	6004 (55.97)	7449 (52.95)	
≥ 2	4672 (18.84)	2311 (21.54)	2361 (16.78)	

^a^ *p* based on chi‐square test or Wilcoxon’s rank sum test, as appropriate.

### 3.2. Three Dietary Patterns

The KMO of the three dietary patterns was 0.876 and *p* value of Bartlett’s tests was less than 0.05. Besides, the three patterns accounted for 51.74% of the variation in dietary consumption and met the retention criteria of eigenvalues higher than 1.0. Dietary pattern A, namely refined grains‐eggs pattern, accounting for 28.58% of the variance, was identified by high factor loadings for refined grains, eggs, fruits, red meats, dairy products, and leafy vegetables. Dietary pattern B, namely nuts‐tubers vegetables pattern, accounting for 15.21% of the variance, was identified by high factor loadings for nuts, tubers vegetables, legumes, whole grains, and aquatic products. Dietary pattern C, namely snacks pattern, accounting for 7.94% of the variance, was identified by high factor loadings for fried and barbecued, fast foods and savoury snacks, SSB, desserts and sweet snacks, and processed meats (Table 2).

**TABLE 2:** Dietary patterns and factor loadings identified using principal component analysis^a^.

Food group	Factor loading
Dietary Pattern A^b^	
Total Variance: 28.58%	
Refined grains	0.710
Eggs	0.705
Fruits	0.678
Red meats	0.669
Dairy products	0.635
Leafy vegetables	0.593
Dietary Pattern B^c^	
Total Variance: 15.21%	
Nuts	0.752
Tubers vegetables	0.714
Legumes	0.688
Whole grains	0.614
Aquatic products	0.572
Dietary Pattern C^d^	
Total Variance: 7.94%	
Fried and barbecued	0.868
Fast foods and savoury snacks	0.780
SSB	0.735
Desserts and sweet snacks	0.659
Processed meats	0.589

*Note:* KMO = 0.876.

^a^Factor loading with absolute values ≥ 0.50 were listed in the table among 17 foods or food groups.

^b^Dietary pattern A was identified by high factor loadings for refined grains, eggs, fruits, red meats, dairy products, and leafy vegetables.

^c^Dietary pattern B was identified by high factor loadings for nuts, tubers vegetables, legumes, whole grains, and aquatic products.

^d^Dietary pattern C was identified by high factor loadings for fried and barbecued, fast foods and savoury snacks, SSB, desserts and sweet snacks, and processed meats.

### 3.3. Association Between Dietary Patterns and Myopia

**FIGURE 2:**
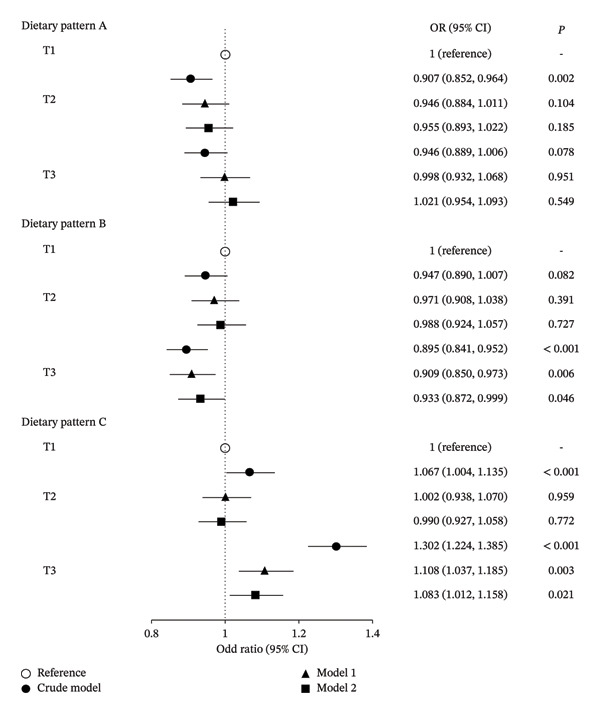
Association between each dietary pattern tertiles and myopia by binary logistic regression. Crude model: unadjusted; model 1: adjusted for age, sex, region, and parental myopia; model 2: additionally adjusted for homework time and outdoor exercise time. Dietary pattern A, i.e., refined grains‐eggs pattern, was identified by high factor loadings for refined grains, eggs, fruits, red meats, dairy products, and leafy vegetables; dietary pattern B, i.e., nuts‐tubers vegetables pattern, was identified by high factor loadings for nuts, tubers vegetables, legumes, whole grains, and aquatic products; dietary pattern C, i.e., snacks pattern, was identified by high factor loadings for fried and barbecued, fast foods and savoury snacks, SSB, desserts and sweet snacks, and processed meats.

The results of the association between three dietary patterns and myopia are shown in Figure 2. For dietary pattern A (i.e., refined grains‐eggs pattern), participants in the middle tertiles of adherence had a significantly reduced risk of myopia, compared with those in the least adherent reference category (OR = 0.907, 95% CI: 0.852 to 0.964, *p* = 0.002). However, this association was not significant in the adjusted models. For dietary pattern B (i.e., nuts‐tubers vegetables pattern), the highest tertiles of adherence were associated with decreased risk of myopia, compared with the least adherent reference category (OR = 0.895, 95% CI: 0.841 to 0.952, *p* < 0.001). The results remained significant after adjusting for age, sex, region, parental myopia, homework time, and outdoor exercise time (OR = 0.933, 95% CI: 0.872 to 0.999, *p* = 0.046). For dietary pattern C (i.e., snacks pattern), characterized by high consumption of fried and barbecued, fast foods and savoury snacks, SSB, desserts and sweet snacks, and processed meats, the highest tertiles of adherence was a risk factor for myopia. The association of adherence to snacks pattern with myopia was significant in the crude model (OR = 1.302, 95% CI: 1.224 to 1.385, *p* < 0.001) and adjusted model 1 (OR = 1.108, 95% CI: 1.037 to 1.185, *p* = 0.003) and 2 (OR = 1.083, 95% CI: 1.012 to 1.158, *p* = 0.021).

## 4. Discussion

In this cross‐sectional study of 24,797 children and adolescents, three distinct dietary patterns were identified from the FFQ data: the refined grains‐eggs pattern (dietary pattern A), the nuts‐tubers vegetables pattern (dietary pattern B), and the snacks pattern (dietary pattern C). Adjusting for age, sex, region, parental myopia, homework time, and outdoor exercise time, the highest tertiles of adherence to nuts‐tubers vegetables pattern were linked with a decreased risk of myopia compared to the least adherent reference category. This pattern was characterized by nuts, tubers vegetables, legumes, whole grains, and aquatic products. The highest tertiles of adherence to snacks pattern were linked with an increased risk of myopia compared to the least adherent reference category. This pattern was characterized by fried and barbecued, fast foods and savoury snacks, SSB, desserts and sweet snacks, and processed meats. In contrast, the refined grains‐eggs pattern, characterized by refined grains, eggs, fruits, red meats, dairy products, and leafy vegetables, was not associated with myopia.

The nuts‐tubers vegetables pattern and the snacks pattern showed considerable overlap in the features of these items with the components of other theoretically healthy dietary patterns. Both dietary patterns are similar to the consumption of foods found in the Planetary Health Diet and Dietary Approaches to Stop Hypertension (DASH) Diet. The Planetary Health Diet consists of whole grains, vegetables, fruits, legumes, nuts, and unsaturated oils; includes a low to moderate amount of seafood and poultry, and includes no or a low quantity of processed meat, red meat, added sugar, and starchy vegetables [9]. The DASH diet emphasizes vegetables, fruits, whole grains, low‐fat dairy products, poultry, fish, legumes, and nuts; it includes no or a low quantity of red meats, desserts, and SSB [10]. Both dietary patterns share several common features, including reduced consumption of refined grains, processed foods, red meats (protein comes more from white meats, legumes, and nuts), and added sugars, high consumption of whole grains, potatoes which are rich in dietary fiber, as well as fruits and vegetables, moderate consumption of aquatic products and dairy products. A recent cohort study showed that the DASH diet was associated with a decreased risk of incident type 2 diabetes [11]. Hyperglycemia can also have an adverse effect on vision.

To date, few studies have investigated the potential influence of dietary patterns on myopia. A study by Yin et al. reported that dietary patterns characterized by high consumption of meats, aquatic products, dairy and its products, eggs, legumes, vegetables, fruits, grains, and potatoes were associated with a lower risk of myopia [12]. Consistent with Yin et al.’s study, our study also showed the link between the dietary patterns characterized by high consumption of whole grains, vegetables, aquatic products, legumes, and myopia. In addition, our study suggested the dietary pattern, including high consumption of fried and barbecued, fast foods and savoury snacks, SSB, desserts and sweet snacks, and processed meats, increased the risk of myopia. Notably, leafy vegetables and tuber vegetables were investigated separately in our study. Phenolic compounds, carotenoids, and other bioactive compounds in tubers vegetables presented high antioxidant and hypoglycemic [13]. Purple potatoes, as one of the tubers vegetables, are rich in anthocyanins, inhibiting myopia’s occurrence and development. Myopia inhibition is possibly achieved by relaxation of ciliary smooth muscle and modulating lens refraction [14].

Existing evidence revealed the relationship between specific foods and myopia. Evidence from 12,397 Chinese children aged 11 to 14 reported that the intake of sugary food, including cakes, candies, preserved fruits, chocolates, and ice cream, had a positive correlation with the risk of myopia [15]. Similarly, our study also suggested snacks pattern consisted of desserts and sweet snacks, and SSB was linked with a greater possibility of myopia. Long‐term excessive sugar intake can influence blood glucose levels, insulin, and glucagon [16]. Elevated blood glucose levels and abnormal glucose metabolism may affect the polyol and acetylcholine signaling pathways, potentially inducing refractive and axial myopia [15, 17]. A cross‐sectional study involving 180 children indicated that refined grains consumption increased the probability of myopia for girls but decreased it for boys [18]. However, due to the small sample size, larger cohort studies are needed to confirm these findings. Our study found no association between the refined grains‐eggs pattern and myopia.

The strengths of this study include its large sample size and citywide scale. This study also utilized comprehensive evaluations of dietary factors rather than single food and observed the associations of nuts‐tubers vegetables pattern and snacks pattern with risk of myopia, which provides crucial views for the primary prevention of myopia through dietary adjustment among children and adolescents. However, several limitations need to be addressed. First, causal inferences cannot be addressed due to the cross‐sectional design. Second, FFQ is susceptible to recall bias and measurement errors from the absence of certain food items, although a wide range of food groups have been assessed. Additionally, refraction without cycloplegia may overestimate the myopia rate in children. Because of the huge number and considering the feasibility of the survey, UCVA and noncycloplegia refraction were assessed during ocular examinations. It has been reported that a higher sensitivity of UCVA combined with noncycloplegia refraction is achieved than with either of the two tests alone (UCVA: 63.55%; noncycloplegia refraction: 78.50%; the combination test: 84.35%) [19]. Hence, future prospective studies with cycloplegic refraction incorporating more accurate evaluations of dietary and other potential factors are warranted to verify the conclusions further.

In summary, this study identified three dietary patterns in children and adolescents: the refined grains‐eggs pattern, nuts‐tubers vegetables pattern, and snacks pattern. Nuts‐tubers vegetables pattern, characterized by nuts, tubers vegetables, legumes, whole grains, and aquatic products, decreased the risk of myopia. The snacks pattern, characterized by fried and barbecued, fast foods and savoury snacks, SSB, desserts and sweet snacks, and processed meats, increased the risk of myopia. Therefore, modifying the dietary patterns could be a potential public health measure against myopia, providing new prospects for primary prevention of myopia.

## Author Contributions

Tongtong Li, Jing Yang, and Jing Yan contributed equally to the paper.

## Funding

This research was funded by the National Key Research and Development Program of China (Grant Number 2021YFC2401404), National Natural Science Foundation of China (Grant Numbers 82020108007, 81830026), and Major Social Science Research Project of Tianjin Municipal Education Commission (Grant Number 2023JWZD10).

## Conflicts of Interest

The authors declare no conflicts of interest.

## Data Availability

The data that support the findings of this study are available on request from the corresponding authors. The data are not publicly available due to privacy or ethical restrictions.
